# Targeting Pro-Inflammatory Interleukin-1 Beta Signaling to Modulate Neuroinflammatory Processes Associated with Cognitive Decline in Dementia with Lewy Bodies

**DOI:** 10.3390/cells15191803

**Published:** 2026-10-02

**Authors:** John J. Alam, Ursula A. Germann, Paul Maruff

**Affiliations:** 1 CervoMed, Inc., Boston, MA 02116, USA; ugermann@eippharma.com; 2 Cogstate, Ltd., Melbourne 3000, Australia; pmaruff@cogstate.com; 3 Alzheimer’s Disease Research Group, Florey Institute for Neuroscience and Mental Health, Melbourne 3010, Australia

**Keywords:** neuroinflammation, dementia with Lewy bodies, interleukin-1 beta, cognitive decline, neflamapimod, p38 MAP kinase, p38α

## Abstract

**Highlights:**

**What are the main findings?**

- The oral p38α kinase inhibitor neflamapimod potently (EC50 ~6 nM) inhibits IL-1β-induced IL-6 and IL-8 production from human peripheral blood mononuclear cells.
- In clinical studies, neflamapimod reduces CSF levels of IL-6 and IL-8 in patients with early Alzheimer’s disease, and in patients with dementia with Lewy bodies (DLB) increases the β-amyloid peptide 1–42 to 1–40 (Aβ42/40) ratio in plasma, while reducing plasma glial fibrillary acidic protein (GFAP) levels.

**What is the implication of the main finding?**

- Neflamapimod demonstrated potent inhibition of pro-inflammatory IL-1β signaling in vitro at clinically relevant drug concentrations.
- The associated effects on CSF cytokines, plasma Aβ42/40 ratio, and plasma GFAP in clinical studies support the hypothesis that inhibition of p38α-dependent IL-1β signaling beneficially modulates neuroinflammatory processes underlying cognitive decline in DLB, and potentially in AD.

**Abstract:**

Interleukin-1 beta (IL-1β) is implicated as a molecular driver of cognitive decline in Alzheimer’s disease (AD) and dementia with Lewy bodies (DLB), potentially through activation of pro-inflammatory pathways within the neuron that impair synaptic function. Because pro-inflammatory IL-1β signaling depends on p38α kinase, the oral brain-penetrant p38α inhibitor neflamapimod has the potential to reverse synaptic dysfunction and treat cognitive decline in these contexts. Neflamapimod has also shown positive effects in animal models and in clinical studies in AD and DLB. Herein we evaluated neflamapimod effects on pro-inflammatory IL-1β signaling in vitro and in patients with AD or DLB. In human PBMCs, neflamapimod inhibited IL-1β-induced IL-6 and IL-8 production with EC50 values of 6.3 ± 1.3 nM and 7.1 ± 3.2 nM, respectively. In AD, six weeks of neflamapimod 40 mg BID treatment reduced CSF levels of IL-6 and IL-8, with maximal effects at trough drug concentration ≥4 ng/mL (9 nM). In DLB, neflamapimod 40 mg TID increased the plasma Aβ42/40 ratio and reduced plasma GFAP levels, treatment effects consistent with reduced pro-inflammatory IL-1β signaling. Collectively, these results support the hypothesis that inhibition of p38α-dependent IL-1β signaling may beneficially modulate neuroinflammatory processes that otherwise are associated with cognitive decline in DLB, and potentially in AD.

## 1. Introduction

Converging evidence indicates interleukin-1 beta (IL-1β) to be a key molecular driver of cognitive decline in a range of neurologic disorders [1,2,3], including Alzheimer’s disease (AD) and dementia with Lewy bodies (DLB) [4,5]. For example, polymorphisms in the gene for IL-1β have been linked to age-related cognitive decline [6], as well as AD [7], while increased levels of IL-1β have been documented extensively in older adults with both AD and DLB. Mechanistically, IL-1β inhibits the formation of long-term potentiation (LTP) [8,9] and, by inhibiting choline acetyltransferase expression, impairs the function of acetylcholine-producing neurons (cholinergic neurons) in the basal forebrain [10,11], dysfunction and degeneration of which are a major driver of disease expression in DLB [12], as well as in a subset of AD [13]. However, IL-1β may also be neuroprotective or exert pro-cognitive effects [14,15,16], observations which may have contributed to the lack of success to date of drug-development efforts targeting IL-1β to reduce cognitive decline, as well as of other anti-inflammatory approaches to treating neurodegeneration.

One novel approach to specifically targeting the deleterious effects of IL-1β, while preserving its putative neuroprotective effects, may be to inhibit signaling via the classic pro-inflammatory IL-1 receptor accessory protein (IL-1RAcP) [1,9,17], expressed on immune cells and most non-neuronal cells, while not inhibiting signaling via the neuron-specific IL-1 receptor (IL-1RAcPb) [17], through which neuroprotective effects are proposed to be mediated [1,9,18]. Pro-inflammatory IL-1β signaling via IL-1RAcP depends on p38α kinase while IL-1RAcPb signaling (i.e., signaling via the neuronal isoform) does not and therefore the oral brain-penetrant p38α inhibitor neflamapimod has the potential to block harmful IL-1β effects while preserving its beneficial signaling within neurons. Indeed, when evaluated in aged rats, the positive effects in the Morris-Water-Maze test were seen at a dose and plasma drug concentration associated with inhibition of IL-1β signaling and not at doses where IL-1β levels were reduced [19]. Neflamapimod has also demonstrated beneficial effects on basal forebrain cholinergic degeneration and behavioral outcomes in animal models [20], and published clinical studies have demonstrated target engagement in phase 2 clinical trials in AD [21,22] and clinical proof-of-concept in a phase 2a clinical study in DLB [20]. More recently, the results of a phase 2b clinical study in DLB [22] have been reported at medical conferences [23,24] and neflamapimod is reported to be progressing towards phase 3 development in DLB [25].

Though the scientific rationale for targeting IL-1β signaling with a p38α inhibitor is established, whether this mechanism is relevant to the preclinical and clinical activity of neflamapimod is not known. Herein, we investigated the actions of neflamapimod on pro-inflammatory IL-1β signaling in vitro, and on potential downstream pharmacodynamic consequences of IL-1β activity in data from a yet-to-be reported phase 2a clinical trial AD, as well as associate these data to relevant biomarker data in the phase 2b clinical trial in DLB. Specifically, we assessed effects of neflamapimod on IL-1β-induced IL-6 and IL-8 production in peripheral blood mononuclear cells in vitro, and the effects of six weeks of treatment with oral neflamapimod on IL-6 and IL-8 in the cerebrospinal fluid (CSF) of patients with AD. In addition, we report on the effects of neflamapimod in the recently completed phase 2b clinical trial in DLB on the ratio of β-amyloid peptide 1–42 to β-amyloid peptide 1–40 (Aβ42/Aβ40 ratio) and glial fibrillary acidic protein (GFAP) in plasma, both of which are expected to be influenced by IL-1β-mediated neuroinflammatory activity. Collectively, these findings demonstrate that neflamapimod appears to inhibit pro-inflammatory IL-1β signaling. Furthermore, the evaluation of neflamapimod in the context of preclinical data and clinical results in DLB suggests this mechanism may contribute to the clinical benefits observed in DLB, a disorder in which neuroinflammation-driven dysfunction and degeneration of the basal forebrain cholinergic system are believed to be major contributors to cognitive decline.

## 2. Methods

### 2.1. Participants and Design of Clinical Studies

For the in vitro studies, fresh human whole blood was collected in sterile containers containing K2EDTA to prevent coagulation by Research Blood Components, LLC (Watertown, MA, USA) from different appointed, prescreened human blood donors and delivered to SBH Sciences (Natick, MA, USA) within approximately 2 h after collection via a courier.

Patients with AD were enrolled in Study 303 (NCT02423200), a single-center, 6-week clinical pharmacology study of neflamapimod (then code-named VX-745) in patients with a confirmed diagnosis of mild cognitive impairment (MCI) due to AD or with mild AD (consistent with National Institute of Aging and Alzheimer’s Association research diagnostic guidelines) and a Mini-Mental State Examination (MMSE) score of 17 to 30 (range of enrolled patients). A cerebrospinal fluid (CSF) catheter was placed 24 h prior to the first dose and on Day 41, and cytokines were assessed at 0, 3, 6, 12, 18 and 24 h after catheter insertion; CSF neflamapimod drug concentrations were determined 3 and 6 h after the first dose and the morning dose on Day 41. Full 12 h pharmacokinetic profiles were obtained after the first dose on Day 1 and Day 41, respectively. While data from this study have been presented at a scientific conference (9th Clinical Trials in Alzheimer’s disease (CTAD) conference, San Diego, 8–10 December 2016), the current report represents the first and primary publication of the results from this study.

The patients with DLB were enrolled in the RewinD-LB study (NCT05869669), which was a multi-center phase 2b clinical study of neflamapimod in two parts: Part A: randomized double-blind treatment phase during which participants received either neflamapimod 40 mg TID or matching placebo capsules; Part B, a 32-week open-label extension, during which participants received neflamapimod 40 mg TID. A total of 159 participants with probable DLB by consensus clinical criteria were randomized into Part A, of which 149 entered Part B. To reduce the enrollment of patients with Alzheimer’s co-pathology, individuals with elevated plasma p-tau181 were excluded. The protocol originally used a ≥2.4 pg/mL p-tau181 cutoff, which was based on the literature that reported data [26] correlating the AD pathology status by a PET scan with plasma p-tau181 utilizing the Simoa^®^ pTau-181 V2.0 assay. However, because the V2.0 assay became unavailable at study initiation, the V2.1 assay was used instead, and the exclusion cutoff was adjusted to ≥27.2 pg/mL, based on manufacturer guidance at the time that 27.2 pg/mL in V2.1 corresponded to 2.4 pg/mL in V2.0. The plasma for protein biomarker assessments was obtained at baseline, week 16 (end of Part A and start of Part B), and week 48 (end of Part B). The primary results of this trial have been presented at medical conferences [23,24] and are being published separately.

### 2.2. In Vitro Peripheral Blood Mononuclear Cell (PBMC) Assays

Assays were conducted per Duffy et al. [27]. Human PBMCs were plated in flat-bottom 96-well tissue culture microplates at 4 × 10^5^ cells/well in 100 µL RPMI-1640 medium supplemented with 10% fetal bovine serum (FBS) and 1× penicillin/streptomycin. Neflamapimod dissolved in dimethyl sulfoxide (DMSO) was prepared as 3-fold serial dilutions in tissue culture medium at 4× the desired final compound test concentrations and at 4× the final DMSO concentration of 0.1%, and 50 µL/well was added to the PBMC cultures. Following a 15 min incubation at 37 °C and 5% CO_2_, 50 µL/well stimulant was added at 4× the final concentration in tissue culture medium to yield a total assay volume of 200 µL/well. Recombinant human IL-1β (PeproTech, now ThermoFisher Scientific, Waltham, MA, USA, Cat. # 200-01) was added at a 0.5 ng/mL final concentration, whereas LPS (Sigma, St. Louis, MO, USA, Cat. #2262) was used at a final concentration of 4 ng/mL. After 16 h incubation at 37 °C and 5% CO_2_, the cell microplates were centrifuged at 300× *g* and culture supernatants were collected for Enzyme Linked Immunosorbent Assay (ELISA) analysis. Determinations of IL-1β-stimulated human IL-6 and IL-8 in PBMC culture supernatants were performed using the following ELISA kits: PeproTech (Cranbury, NJ, USA) EDK Human IL-6 ELISA Kit, Cat. # 900-K16, R&D Systems (Minneapolis, MN, USA) DuoSet Human IL-8 ELISA Kit, Cat. # DY208, PeproTech EDK Human IL-8 ELISA Kit, Cat. # 900-T18. Determinations of LPS-induced human IL-1ß and TNFα in PBMC culture supernatants were performed using the following ELISA kits: R&D Systems DuoSet Human IL-1ß ELISA Kit, Cat # DY201 and PeproTech EDK Human TNFα ELISA Kit, Cat. # 900-K25.

To determine the cell viability of the remaining PBMCs, 20 µL of Promega Substrate Cell Titer 96^®^ AQ_ueous_ One Solution Reagent (Promega (Madison, WI, USA), Cat. # G358B) were added to each well of the original cell plate. The One Solution Reagent contained a tetrazolium compound [3-(4,5-dimethylthiazol-2-yl)-5-(3-carboxymethoxyphenyl)-2-(4-sulfophenyl)-2H-tetrazolium, inner salt; MTS] and an electron coupling reagent (phenazine ethosulfate; PES). PES has enhanced chemical stability, which allowed it to be combined with MTS to form a stable solution. Then the microplate was incubated at 37 °C and 5% CO_2_ and absorbance at 490 nm was measured at every hour with a 96-well plate reader. The quantity of the formazan product as measured by the optical density at 490 nm (OD_490_) was directly proportional to the number of living cells in the culture. Once the OD_490_ reached OD 1.000 or above, plate reading was stopped and the final reading was used to determine the cell viability. This amount of signal generally required approximately four hours.

PBMC assays and analysis of the data were conducted at SBH Sciences, Natick, MA, USA.

### 2.3. CSF and Protein Biomarker Assessments

Cytokine measurements by ELISA in CSF were collected to assess the balance of pro-inflammatory versus anti-inflammatory cytokines. The t-helper type 1(TH1)/t-helper type 2 (TH2) multiplex cytokine (interferon gamma, IL-1β, IL-2, IL-4, IL-5, IL-8, IL-10, IL-12 protein 70, IL-13, TNFα) panel by Mescoscale Discovery (MSD) was originally utilized. The analysis was conducted at Synexa, Cape Town, South Africa, at the time that the clinical Study 303 was originally conducted. Of the cytokines analyzed, only IL-8 levels were measurable in more than 50% of samples (levels for IL-8 were greater than lower limit of quantification, LLOQ, in every sample) and accordingly only those levels are reported from this original panel (note: for IL-1β, every sample was below LLOQ, 4.3 pg/mL). IL-6 levels were evaluated at a later point in time, also using the MSD platform, at the clinical chemistry laboratory at Amsterdam Medical Center (AMC), Amsterdam, the Netherlands. The AMC also measured Aβ42 and Aβ40 levels in the plasma using the Simoa Neurology 4-PLEX E (N4-PE) Advantage kit on the Simoa platform (Quanterix, Billerica, MA, USA). The plasma glial fibrillary acidic protein (GFAP) and neurofilament light chain (NfL) results are being reported separately with the main clinical trial results of the RewinD-LB study.

### 2.4. Pharmacokinetics

Neflamapimod drug concentrations in plasma and CSF were determined at Charles River Laboratories (CRL) Edinburgh, UK, utilizing a Liquid Chromatography–Mass Spectrometry/Mass Spectrometry (LC-MS/MS) assay validated at CRL. The LLOQ was 1.5 ng/mL in plasma and 0.5 ng/mL in CSF.

### 2.5. Statistical Analyses

PBMC assay ELISA data analysis was performed using an SBH Sciences in-house Excel template to generate concentration-response curves. A four-parameter logistic (4PL) fit of the standard curve was calculated utilizing Sigma Plot version 12.5 and the resulting standard curve fit for each cytokine was used to determine the concentrations of the respective cytokines in the samples. To determine EC_50_, a non-linear regression analysis was performed for the Log10 [neflamapimod concentration] versus response and the half-maximal effective concentration (EC50) for every individual volunteer for IL-6 (*N* = 3) and IL-8 (*N* = 5) was determined based on the 50% inhibition of IL-1β-stimulated cytokine production using a variable slope, 4-parameter logistic curve fit. The EC_50_ values for the individual volunteers were averaged to determine the reported EC_50_, with standard errors, for each of IL-6 and IL-8.

CSF IL-6 and IL-8 data were analyzed with a sigmoidal 4-parameter logistic (4PL) model using GraphPad version 10.4.2 (534) at CervoMed. Plasma Aβ42/40 and GFAP data analyses were conducted at Anoixis Corporation, following a biomarker statistical analysis plan for the RewinD-LB study. The change in plasma Aβ42/40 from baseline to end-of-treatment was evaluated using a paired *t*-test, while because of a skewed distribution, the change in plasma GFAP was evaluated using a nonparametric Wilcoxon signed-rank test.

## 3. Results

### 3.1. Participants Enrolled in Phase 2a Study in Early AD (Study 303)

Nine patients were enrolled. The mean age was 70.8 years, and mean weight was 64.10 kg. Five (55.6%) patients were female and four (44.4%) were male. Most of the patients were Asian (six [66.7%]), while two (22.2%) were Black or African American, and one (11.1%) was White. The mean (median) CDR-SB score for all patients was 0.60 (0.50), mean (median) HVLT-R Total Recall score was 19.4 (20.5) and mean (median) MMSE score was 25.9 (26.0). One subject discontinued within the first week of dosing (see safety), and the remaining eight subjects completed dosing as scheduled. Of the eight subjects who completed treatment, seven had complete sets of CSF samples at baseline and on Day 40; in one subject, the CSF catheter became blocked after the three-hour sample collection.

### 3.2. CSF Drug Concentrations in Patients with AD (Study 303)

Neflamapimod drug concentrations in CSF were detectable in all post-dosing samples in all subjects in Study 303 but were quantifiable in four subjects across a total of 12 time-points. The mean (SD) concentration was 0.874 (0.406) ng/mL in the twelve samples. The corresponding simultaneously obtained mean (SD) plasma drug concentration was 14.96 (7.81) ng/mL and the mean (SD) CSF-to-plasma ratio was 0.062 (0.01). That is, neflamapimod drug concentration in CSF was 6.2% of simultaneously measured plasma drug concentration, similar to that observed in a 14-day pharmacokinetic study in dogs, where at both acute (after the first dose) and steady-state time-points, the neflamapimod concentration in the brain was 1.7-fold that in the plasma at matched time-points [15].

### 3.3. Inhibition of IL-1β and LPS Signaling in Human Peripheral Blood Mononuclear Cells

All assay conditions were tested in triplicate. DMSO alone at a 0.1% concentration, i.e., a sample containing 0 nM test compound, served as a test article-negative control (i.e., vehicle control) for each assay series in the absence or presence of optimized concentrations of 0.5 ng/mL recombinant human IL-1ß, or of 4 ng/mL bacterial LPS and in the absence or presence of a titration of neflamapimod. Unstimulated versus vehicle-treated, as well as neflamapimod-only controls were only assessed during the PBMC assay development phase and no effects on cytokines or cell viability were detectable. The results for the cytotoxicity assays measuring mean values for the relative cell viability resulting from the various treatments indicated that none of the experimental conditions resulted in obvious cytotoxicity, defined as a relative mean cell viability of <80%. IL-1ß was clearly required for IL-6 or IL-8 production since negative controls containing the DMSO vehicle, but no IL-1ß, showed levels of IL-6 (pg/mL) or IL-8 (pg/mL) below or near the lower limit of quantitation (LLOQ). Similarly, in all PBMC assays, LPS was required to stimulate IL-1ß or TNFα production. Negative controls containing DMSO vehicle, but no LPS, showed no detectable levels of IL-1ß (<LLOQ) or TNFα (<LLOQ).

**Figure 1:**
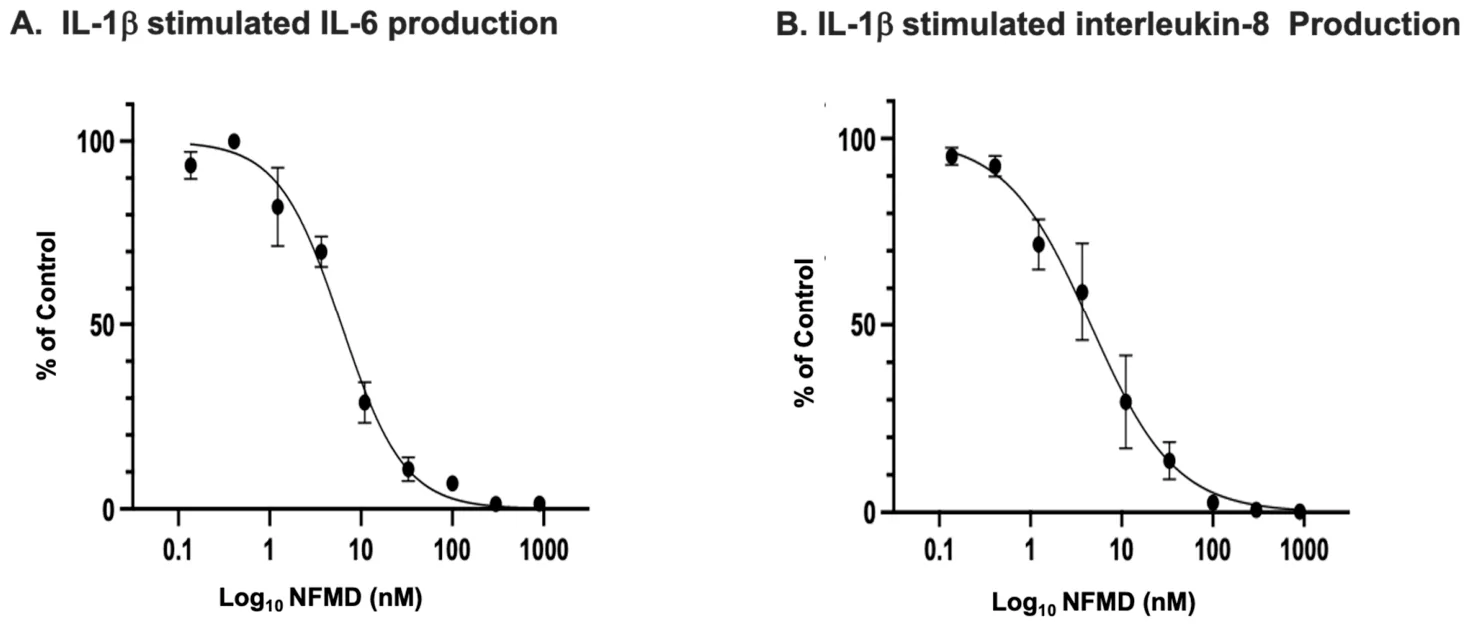
Effect of neflamapimod on interleukin-1 beta (IL-1β) signaling in human peripheral blood mononuclear cells. (**A**) Interleukin-6 (IL-6) and (**B**) Interleukin-8 (IL-8) levels in supernatant after 16 h incubation of peripheral blood mononuclear cells (PBMCs) with 0.5 ng/mL IL-1β and indicated concentrations of neflamapimod. EC50 values (mean ± SEM) were 6.3 ± 1.3 nM for IL-1β-induced IL-6 production and 7.1 ± 3.2 nM for IL-1β-induced IL-8 production.

As shown in Figure 1, neflamapimod potently inhibited IL-1β-induced IL-8 and IL-6 production from human PBMCs. EC50 values (mean ± SEM) were 6.3 ± 1.3 nM for IL-1β-induced IL-6 production (evaluated in three donors) and 7.1 ± 3.2 nM for IL-1β-induced IL-8 production (evaluated in five donors). Neflamapimod was less potent in inhibition of LPS signaling, as the EC50 for LPS-stimulated IL-1β production (evaluated in four donors) was 22.1 ± 6.7 nM and that for LPS-stimulated TNF-α production (evaluated in four donors) was 161.7 ± 66.8 nM. Individual donor results and detailed output from non-linear regression analyses are provided in the Supplemental Materials.

### 3.4. Change in CSF Interleukin-6 and Interleukin-8 After Six Weeks of Neflamapimod Administration

**Figure 2:**
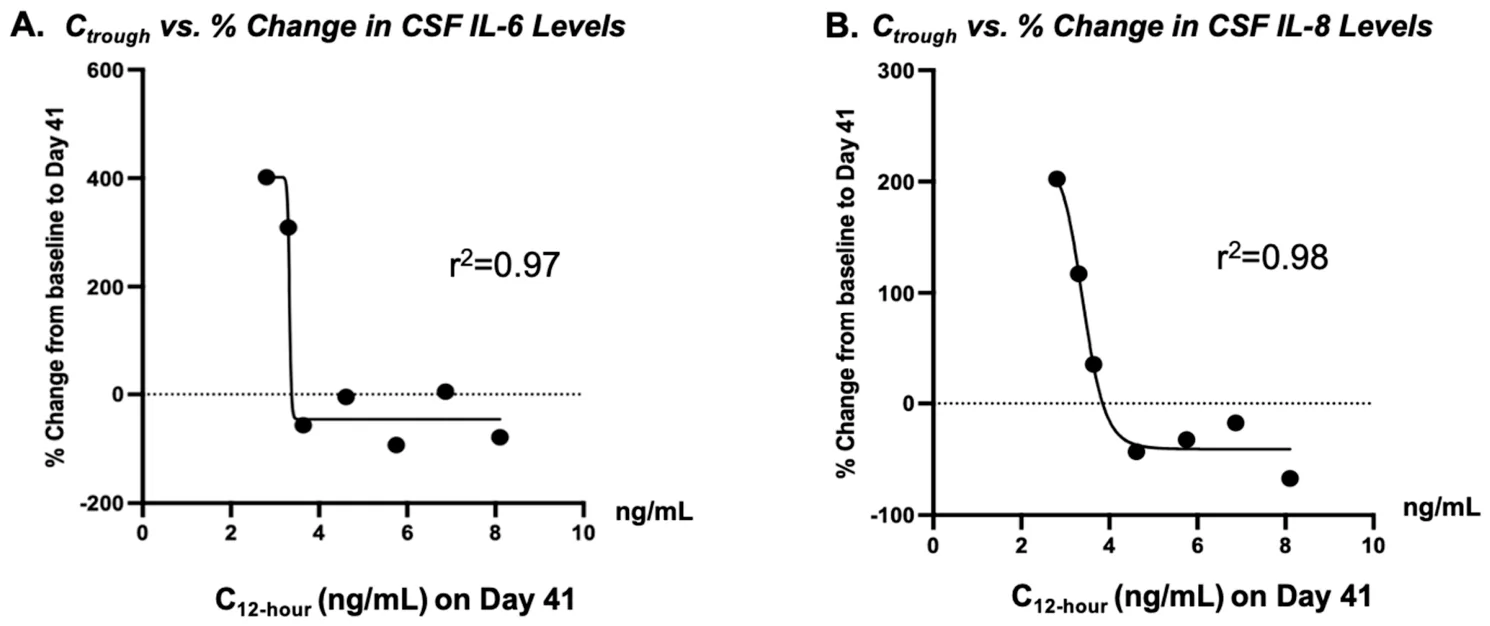
Correlation between plasma drug trough concentration and change in CSF Levels of IL-6 and IL-8 in patients with early Alzheimer’s disease. Correlation between individual subject (*N* = 7) trough (pre-morning dose) plasma drug concentration (C_trough_) level on Day 42 and % Change from Baseline to Day 42 in (**A**) interleukin-6 (IL-6) or (**B**) interleukin-8 levels in cerebrospinal fluid (CSF) in participants with Early Alzheimer’s disease dosed with neflamapimod 40 mg BID for 6 weeks (42 days). To account for circadian changes in levels, a CSF catheter was placed on Day 0 (24 h before commencement of neflamapimod dosing) and start of Day 42, and samples for cytokine assessment were obtained at 0, 3, 6, 12, 18 and 24 h of each 24 h cycle on Day 0 and Day 42. The median value of the six time-points was utilized as the baseline value and the Day 42 value to calculate % change for each participant. r^2^ = 0.97 (standard error of regression, Sy,x, = 50.7%) for the correlation with % change in IL-6 and r^2^ = 0.98 (Sy,x= 21.3%) for % change in IL-8.

For each individual subject, the trough (Day 41 pre-morning dose) plasma drug concentration, C_trough_, versus % change from baseline to Day 41 in CSF levels of IL-8 and IL-6 are shown in Figure 2A,B. In a plasma drug concentration-dependent manner, neflamapimod reduced levels of both cytokines, with an estimated in vivo EC50 of 3.4 ng/mL for CSF IL-8 and 3.3 ng/mL CSF IL-6 (with the number of individuals assessed, 95% confidence interval could not be calculated). For each, the effect appeared to plateau at approximately 4 ng/mL (~9 nM) with no increase in the effect at higher C_trough_ levels.

### 3.5. Plasma Aβ42/40 Ratio and GFAP Levels in the RewinD-LB Clinical Trial

**Figure 3:**
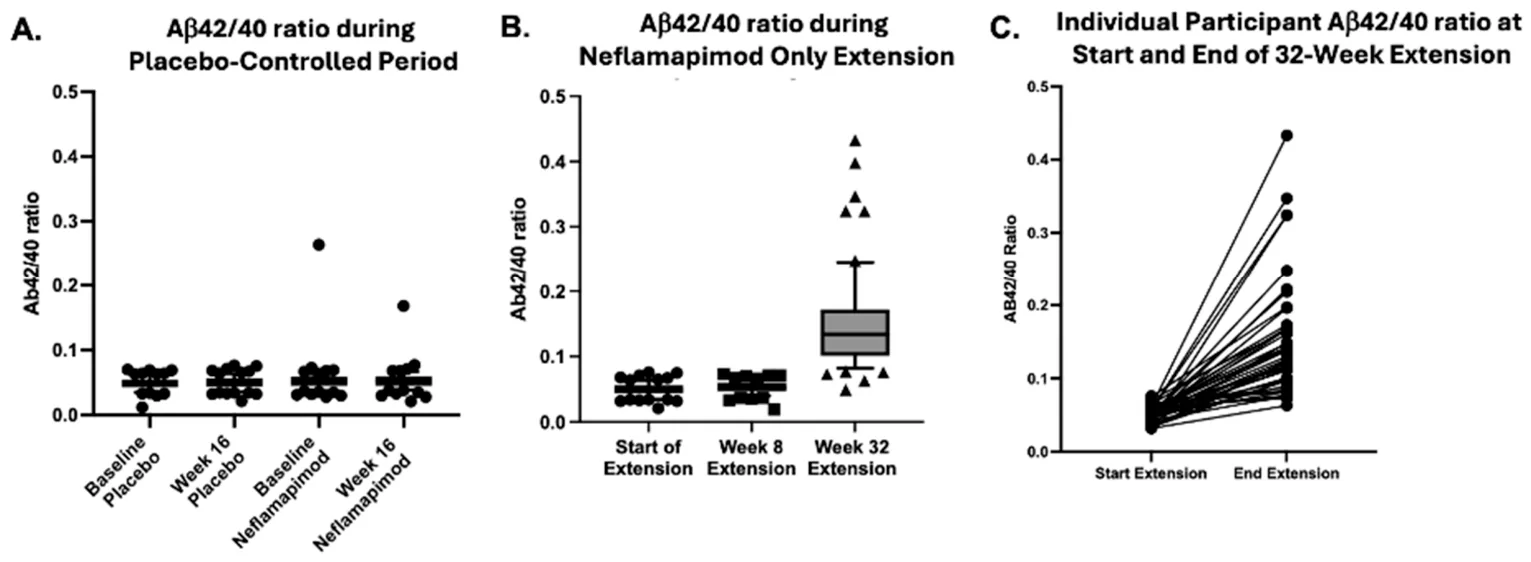
Aβ42/40 ratio during RewinD-LB clinical trial of neflamapimod in patients with dementia with Lewy bodies. Ratio of β-amyloid (1–42) peptide to β-amyloid (1–40) peptide, “Aβ42/40 ratio”, during (**A**) 16-week placebo-controlled phase (*n* = 68 for placebo; *n* = 69 for neflamapimod) and (**B**) 32-week open-label neflamapimod extension phase (*N* = 43) of the RewinD-LB clinical trial of neflamapimod 40 mg TID in patients with dementia with Lewy bodies. There was no difference between the placebo and neflamapimod at either baseline or week 16. Box (median, interquartile range) and whisker (10–90 percentiles) plots. (**C**) Individual participant Aβ42/40 ratio at start of extension and end of extension (within-participant, *N* = 43, comparison of end and start of extension: +0.10, 95% CI: +0.08 to +0.12; paired comparison *p* < 0.0001, paired *t*-test, t = 8.233, df = 42). For the extension phase data presentation, only data from participants who received placebo during the placebo-controlled phase are included. Otherwise, data from all samples analyzed are reported.

Plasma Aβ42/40 ratio is a measure of amyloid aggregation, with a lower ratio indicating greater propensity for amyloid aggregation. Within the RewinD-LB study there was no difference in the plasma Aβ42/40 ratio between placebo recipients and neflamapimod-treated participants during 16-week randomized treatment phase (Figure 3A). However, among participants who received the placebo initially and subsequently crossed over to neflamapimod during the extension phase (*N* = 43), with 32 weeks of treatment (though not with 8 weeks of treatment), from a mean 0.050 (SD = 0.01) at the start of the extension, there was a mean increase of 0.10 in the plasma Aβ42/40 ratio at the end-of-treatment (within-participant comparison, 95% CI: +0.08 to +0.12; paired comparison *p* < 0.0001; paired *t*-test, t = 8.233, df = 42). The increase in the Aβ42/40 ratio was observed consistently across participants (all but two participants showing an increase to >3 SD above the baseline mean; the two who did not, started at 2 SD below the baseline mean), supporting a reproducible pharmacodynamic effect of neflamapimod on this amyloid-related biomarker.

**Figure 4:**
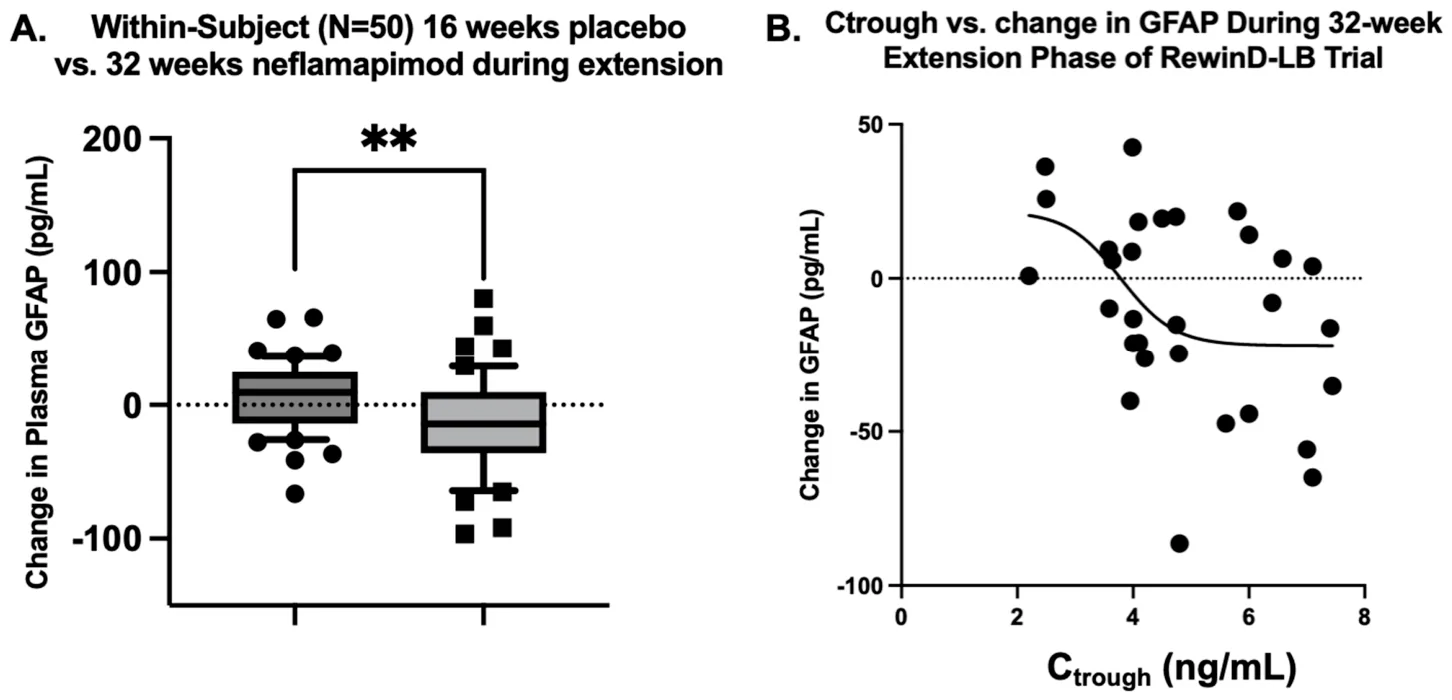
Change in plasma glial fibrillary acidic protein (GFAP) during the open-label extension phase of RewinD-LB study. Change in plasma GFAP during the open-label neflamapimod treatment extension phase of the RewinD-LB clinical study of neflamapimod in patients with dementia with Lewy bodies. (**A**) Within-participant analysis of change in plasma GFAP during the placebo administration period vs. during neflamapimod administration during the extension in participants who received placebo during the placebo-controlled phase and then completed 32 weeks of neflamapimod treatment during the extension (**—*p* = 0.007, Wilcoxon-signed-rank test, sum of signed ranks (W) = −550.0, sum of positive ranks 362.5, sum of negative ranks −912.5; *N* = 50). Box (median, interquartile range) and whisker (10–90 percentiles) plots. (**B**). Relationship between trough plasma drug concentration obtained at week 4 of the extension phase and change in plasma GFAP from start to end of the 32 weeks of the extension phase (*N* = 32). Only data from participants who received placebo during the placebo-controlled phase are included.

Regarding plasma GFAP, in participants who crossed over from the placebo during the randomized period to neflamapimod, during the 32-week extension period, there was a significant reduction in this protein biomarker while receiving neflamapimod (−17.4 pg/mL) compared with both the start of the extension or the change while on placebo (+6.7 pg/mL) [Figure 4A; *p* = 0.006 for change from start of extension to end of extension, Wilcoxon-signed-rank test, sum of signed ranks (W) = −564.0, sum of positive ranks 355.5, sum of negative ranks −919.5; *p* = 0.007, paired within-participant comparison while on neflamapimod vs. while on placebo, Wilcoxon-signed-rank test, sum of signed ranks (W) = −550.0, sum of positive ranks 362.5, sum of negative ranks −912.5]. Because, compared with the treatment effect on the Aβ42/40 ratio, there was greater heterogeneity in the effect on plasma GFAP, an evaluation of the relationship to trough plasma drug concentrations was conducted. As shown in Figure 4B, the relationship between C_trough_ and change in plasma GFAP was like that for CSF IL-6 and IL-8. Note: plasma GFAP results from the placebo-controlled phase of the study, during which there was no evident treatment effect compared to the placebo, will be reported in a separate publication that summarizes the clinical results.

## 4. Discussion

The results of these studies support the hypothesis that the biological changes and therapeutic benefits of treatment arising from neflamapimod in AD and DLB involve inhibition of pro-inflammatory IL-1β signaling. Across the in vitro and biomarker studies, we observed evidence that at clinically relevant plasma drug exposures neflamapimod either directly inhibits pro-inflammatory IL-1β signaling or produces pharmacologic effects consistent with suppression of pathogenic IL-1β signaling

In human PBMC assays, neflamapimod potently inhibited IL-1β-induced IL-6 and IL-8 production, with EC50 values in the low nanomolar range. In contrast, inhibition of upstream LPS-induced cytokine production required substantially higher concentrations, particularly for TNFα. Notably, the potency of neflamapimod against IL-1β signaling closely matched its 9 nM IC50 for p38α inhibition. In contrast, the requirement for a higher concentration of neflamapimod to inhibit LPS-induced cytokine production suggests that inhibition of p38α is not sufficient for this activity. Rather, the EC50 for inhibition of LPS-induced cytokine production approaches the 190 nM IC50 for inhibition of the kinase activity of the beta isoform of p38 (p38β) [27], suggesting that inhibition of p38β may also be necessary to inhibit LPS-induced cytokine production. The involvement of kinase pathways other than p38α and p38β in the activity of neflamapimod with respect to LPS-induced cytokine production is unlikely, as when screened by independent laboratories against 200 human kinases for enzyme inhibition and >400 human kinases for binding, neflamapimod does not inhibit the activity of any kinases other than p38α and p38β [28,29,30].

The differential potency for IL-1β signaling and cytokine production may potentially be important in relation to cognitive decline because of the receptor and cell type specificity of IL-1β signaling in the brain. As discussed previously, while signaling through the canonical IL-1 receptor accessory protein (IL-1RAcP) pathway [9] contributes to synaptic dysfunction, impaired long-term potentiation, and cholinergic neuronal dysfunction, signaling through the neuron-specific IL-1RAcPb isoform may mediate neuroprotective and pro-cognitive effects [17,18]. Because IL-1RAcPb signaling is not dependent on p38α, selective inhibition of p38α at concentrations below those required to suppress IL-1β production would potentially preserve physiological IL-1β levels and beneficial IL-1RAcPb-mediated signaling within neurons while attenuating pathogenic pro-inflammatory signaling.

The CSF biomarker findings in the patients with early AD provide translational evidence that neflamapimod inhibits IL-1β signaling in the CNS. Six weeks of treatment with neflamapimod produced concentration-dependent reductions in CSF IL-6 and IL-8, both downstream mediators of IL-1β signaling, with maximal effects observed at trough plasma concentrations of approximately 4 ng/mL (~9 nM). Notably, this concentration corresponds closely to the in vitro potency of neflamapimod for inhibition of IL-1β signaling, supporting the conclusion that the in vivo effect is mediated through the same mechanism identified in PBMCs.

The CSF pharmacokinetic data reported herein further support this interpretation. In a 14-day pharmacokinetic study in dogs, where at both acute (after the first dose) and steady-state time-points, the neflamapimod concentration in the brain was 1.7-fold that in the plasma at matched time-points, while the CSF levels were approximately 6% of that in the plasma at matched time-points [28]. Based on the current findings that the neflamapimod drug concentration in CSF in patients was 6.2% of simultaneously measured plasma drug concentration, brain concentrations in humans are estimated to be the same as those in dogs, i.e., approximately 1.7-fold higher than plasma concentrations. Assuming this estimate, the plateau in cytokine suppression observed at plasma concentrations of approximately 4 ng/mL would correspond to estimated brain concentrations of ~7 ng/mL (~16 nM), a concentration that is near the EC80 and on the lower plateau of the in vitro concentration-response curve. This concordance between in vitro and in vivo pharmacology suggests strongly that the pharmacodynamic effects captured by reductions in CSF IL-6 and IL-8 reflect inhibition of IL-1β signaling. The plateauing of cytokine suppression above this exposure level further suggests that doses achieving trough plasma concentrations greater than 4 ng/mL are likely to produce near-maximal target engagement, with higher exposures yielding diminishing returns in the inhibition of IL-1β signaling, and its downstream consequences.

The biomarker findings from the RewinD-LB study provide further evidence that neflamapimod modulates relevant neuroinflammatory processes in vivo. First, neflamapimod treatment increased the plasma Aβ42/Aβ40 ratio during the extension phase in participants initially assigned to the placebo. Because lower Aβ42/Aβ40 ratios are associated with increased amyloidogenic processing of amyloid precursor protein (APP) and prior evidence linking neuroinflammation to increased BACE1 activity [31], as well as IL-1β signaling to enhanced amyloidogenic processing [32,33,34,35], the increase in the plasma Aβ42/Aβ40 ratio is consistent with a downstream effect of neflamapimod-mediated inhibition of IL-1β signaling in vivo. In addition, the demonstrated effect on plasma GFAP, a marker of astrocyte activation, is also consistent with neflamapimod treatment reducing neuroinflammatory signaling in the brain. Of note, plasma/serum levels of GFAP are correlated with tau pathology in brain [36,37], which is downstream of IL-1β signaling [7,38]. Moreover, the PK-PD analysis of the plasma GFAP results is similar to the PK-PD analysis of the CSF IL-6/8 data, with a Ctrough of ~4 ng/mL being a threshold below which a reduction in plasma GFAP levels was not evident.

The Aβ42/40 findings also provide potential evidence of target engagement. In transgenic AD mouse models, neuron-specific deficiency of p38α, achieved through knockdown or knockout of MAPK14, reduces BACE1 expression and ameliorates amyloid pathology [39,40,41,42]. Against this mechanistic background, the consistent increase in Aβ42/40 across essentially all neflamapimod-treated participants, approaching three-fold in magnitude, provides strong evidence of a pharmacodynamic effect consistent with neuronal p38α inhibition.

The findings from this *post hoc* analysis of inflammatory factors in the neflamapimod clinical trials program may be particularly relevant for DLB. Although DLB is characterized primarily by α-synuclein pathology, aggregated and oligomeric α-synucleins are known to trigger neuroinflammatory responses, and elevations in IL-1β have been consistently reported in patients with DLB, both in the central nervous system and in the periphery [4,43,44]. Moreover, a recent large-scale proteomic study [45] involving approximately 5700 CSF and plasma samples from patients with neurodegenerative diseases identified IL-6 and IL-1β signaling downstream of fibroblast growth factor receptor (FGFR) signaling as the molecular pathway that was the most important DLB-specific pathogenic pathway. Of note, this most recent finding is also consistent with an emerging consensus around the role of neuroinflammation in the degeneration of basal forebrain cholinergic neurons, which is a central pathogenic feature of DLB. Loss in basal forebrain cholinergic neurons is strongly associated with attentional impairment, cognitive fluctuations, and global cognitive decline in DLB. For example, acute and chronic LPS administration in animal models leads to basal forebrain cholinergic dysfunction and degeneration, respectively [10]. In addition, chronic expression of IL-6 in the brain in transgenic mice (mice expressing interleukin-6 under the GFAP promoter) led to a significant decrease in chothe linergic cell number and age-dependent alterations in the electrophysiological properties of cholinergic neurons [46]. Further, IL-1β signaling leads to α-synuclein overexpression and aggregation [47,48], which otherwise has been reported to contribute to basal forebrain cholinergic degeneration [49]. Consistent with the role of neuroinflammation in basal forebrain cholinergic degeneration and the potential of neflamapimod to modulate IL-1β-driven neuroinflammation, neflamapimod reversed both basal forebrain cholinergic degeneration and associated deficits in behavioral tests in Down syndrome (DS) mice [20]. Importantly, chromosome 21, trisomy of which leads to DS, in addition to containing the gene for amyloid precursor protein, contains the pro-inflammatory S100B gene, and DS is associated with elevated IL-1β levels and neuroinflammation [50]. In line with the clinical evidence of a pro-inflammatory state in individuals with DS, in the preclinical studies conducted with neflamapimod, the levels of phosphorylated p38α and its downstream targets were elevated in trisomic (DS) mice, compared to wild-type mice, and treatment with neflamapimod reduced the phosphorylation state of these proteins to wild-type levels [20].

There is now increasing evidence implicating neuroinflammation, and specifically IL-1β signaling, as an important contributor to neurodegenerative progression within the basal forebrain cholinergic system, a critical pathogenic driver of disease expression and progression in DLB. α-Synuclein pathology activates microglia and inflammatory cascades [51], while inflammatory signaling can further promote synaptic dysfunction and neurodegeneration, creating a self-reinforcing pathogenic cycle. Within this literature context, the current results suggest that inhibition of p38α-dependent IL-1β signaling may interrupt this cycle. Of note, the PK-PD relationships for the clinical effects in AD and DLB are in line with those we report for CSF and plasma biomarkers in the current study, e.g., in both diseases, there is a C_trough_ threshold for clinical activity of approximately 4 ng/mL [20,22,52].

Our findings may also inform on the potential of neflamapimod as a treatment for AD, as IL-1β has been implicated in the pathogenesis of AD [4,50]. While in a 161-patient, 24-week study in early AD, neflamapimod did not demonstrate significant improvement on the primary endpoint (Hopkins Verbal Learning Test), pre-defined PK-PD analyses suggested this was primarily due to the dose being too low (40 mg BID vs. the 40 mg TID that has shown clinical activity in DLB) and there was evidence of clinical activity in patients in the upper quartile of trough plasma drug concentrations (cutoff for upper quartile was 5.1 ng/mL) [22]. Of note, more recent PK-PD analyses of the combined DLB and AD datasets indicate clinical activity against the primary endpoint in the 24-week study in early AD was only evident in participants whose C_trough_ was above 4 ng/mL [52], i.e., aligned with the in vitro potency for inhibition of IL-1β signaling, as well as the effects on CSF IL-6 and IL-8 in patients with early AD, that we report in the current report.

Two observations in this series of studies were inconsistent with the main conclusion and warrant consideration. First, in the AD participants whose C_trough_ concentration was <4 ng/mL there was evidence of an increase in the CSF IL-6 and IL-8 data after six weeks of neflamapimod treatment. One likely explanation for this is the known increase in inflammation with repeated, closely spaced lumbar puncture [53], with CSF neurofilament light chain in primates remaining elevated at least 30 days after an initial lumbar puncture [54,55]. Any such effects would potentially be exaggerated in Study 303, as the initial CSF assessment involved the placement of an indwelling catheter and the withdrawal of ~115 mL of CSF over 24 h. In addition, there is evidence that a sub-optimal dose of neflamapimod may increase CSF GFAP levels [56]. The second potentially inconsistent finding relates to the observation that plasma Aβ42/40 levels did not increase after 16 weeks of neflamapimod treatment but did increase substantially after 32 weeks of treatment. This temporal course suggests that the effect on plasma Aβ42/40 is indirect, rather than directly related to p38α inhibition and the consequent effects on IL1β signaling. One potential explanation for this apparent lack of effect at the early time-points may relate to an effect on microglial p38α that would decrease clearance of oligomeric amyloid beta from the interstitial space [57], an effect that would offset any effect on amyloid beta production on neurons. Such an offsetting effect was observed at plasma drug concentrations similar to those observed with 40 mg TID in a prior study of neflamapimod that assessed effects on amyloid PET scans in patients with early AD [21]. Importantly, such anti-inflammatory effects with p38α inhibition are generally transient over 12–24 weeks [58], potentially due to the redundancy of the anti-inflammatory pathways with the beta isoform, which then would allow for the direct effects of inhibition of neuronal p38α to become apparent at week 32.

Several limitations with the thesis put forward here to explain the benefits neflamapimod should be acknowledged. Regarding the estimated human brain concentrations, they depend on the cross-species assumption that the brain-to-CSF relationship is similar in humans and dogs, an assumption that cannot be directly verified. The biomarker analyses were exploratory and involved relatively small sample sizes. In addition, the CSF cytokine analyses were conducted in patients with AD rather than DLB. Although the mechanistic relevance of IL-1β signaling likely extends across neurodegenerative disorders, differences in immunologic phenotype between these disorders have been reported [45]. Finally, the associations between pharmacological and clinical effects reported here provide indirect evidence that inhibition of IL-1β signaling is the mechanism by which neflamapimod exerts its clinical effects and do not preclude parallel mechanisms that may be involved. Nevertheless, we believe that the convergence of in vitro pharmacology, CSF biomarker effects and plasma biomarker changes, that occur with a consistent PK-PD relationship, when considered in relation to the current understanding of DLB pathogenesis provides a coherent mechanistic framework supporting the clinical observations reported to date with neflamapimod in DLB.

## 5. Conclusions

Neflamapimod demonstrated potent inhibition of pro-inflammatory IL-1β signaling in vitro at clinically relevant drug concentrations. The associated effects on CSF cytokines, plasma Aβ42/40 ratio, and plasma GFAP support the hypothesis that inhibition of p38α-dependent IL-1β signaling may beneficially modulate neuroinflammatory processes that otherwise are associated with cognitive decline in DLB, and potentially in AD.

## Data Availability

The clinical data from RewinD-LB will be made available on Vivli (https://vivli.org) by 12 months from primary publication of the results, which is anticipated by the end of year 2026. The data from Study 303 will be made available upon reasonable request to the corresponding author.

## Supplementary Materials

The following supporting information can be downloaded at: https://www.mdpi.com/article/10.3390/cells15191803/s1, Supplemental Figures and Tables.
