# Supplementary material for: Targeting Pro-Inflammatory Interleukin-1 Beta Signaling to Modulate Neuroinflammatory Processes Associated with Cognitive Decline in Dementia with Lewy Bodies
Source: Cells. 2026 Oct 2;15(19):1803. doi: 10.3390/cells15191803 (PMC13664606; doi:10.3390/cells15191803)
Supplement: Supplementary file 1 [file cells-15-01803-s001.zip › cells-4529873-supplementary.pdf]

Figure S1. Individual Concentration-Response Graph for Neflamapimod Effects on IL-1ß-stimulated IL-6 (Production Represented as % Control)

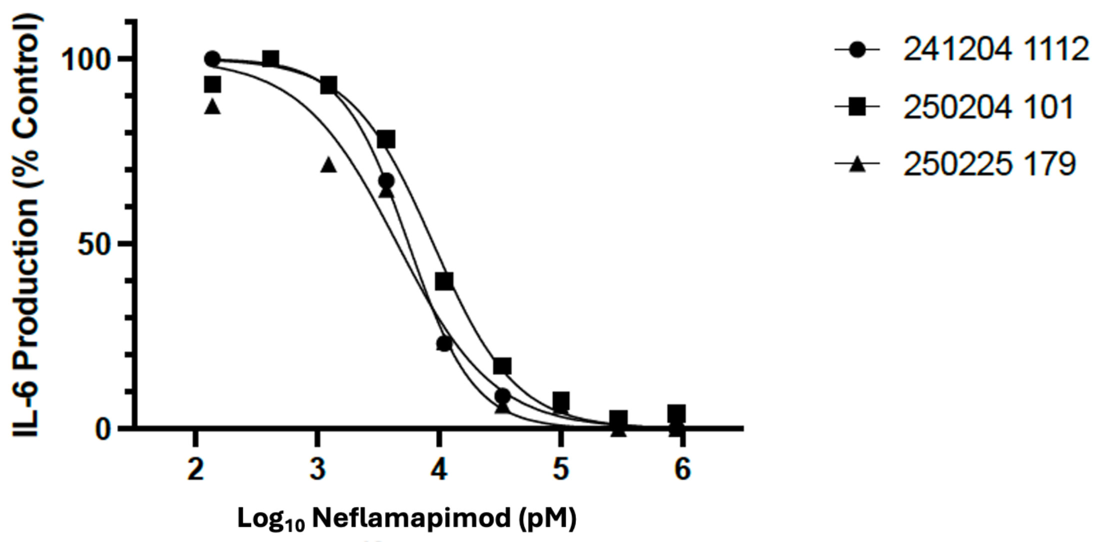

Note: Absolute levels of of IL-1ß-stimulated IL-6 (pg/mL) in the absence of neflamapimod were means ± STD of 184 ± 18, 574 ± 17, and 754 ± 30 for the 3 individual donors.

Figure S2. Concentration-Response Graph for VX-745 Effects on IL-1ß-stimulated IL-8 (Production Represented as % Control)

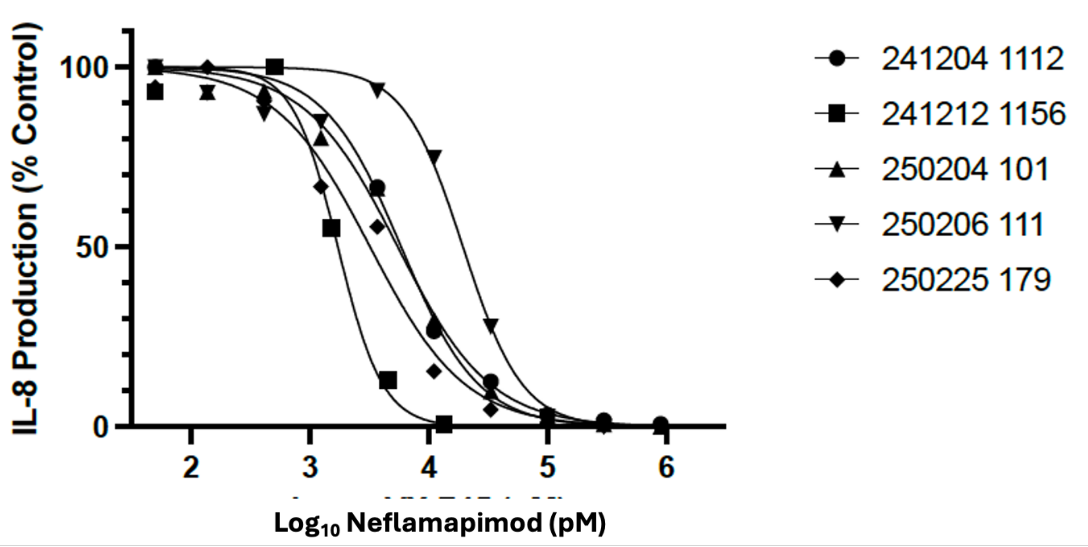

Note: Absolute levels of of IL-1ß-stimulated IL-8 (pg/mL) in the absence of neflamapimod was a mean ± STD of 1652 ± 10, 311 ± 68, 3118 ± 94, 430 ± 82, and 6230 ± 623 for the five individual donors.

Supplemental Table S1: Results for nonlinear regression analysis for neflamapimod and IL-1β-induced IL-8

Log(inhibitor) vs. Normalized Response - Variable Slope - Least Squares Fit	241204 1112	241212 1156	250204 101	250206 111	250225 179
**Best-fit Values**					
LogIC50	3.770	3.236	3.741	4.290	3.507
HillSlope	-1.337	-2.301	-1.117	-1.744	-1.089
IC50	5883	1722	5505	19487	3217
**95% CI (Profile Likelihood)**					
LogIC50	3.702 to 3.833	3.111 to 3.377	3.662 to 3.819	4.122 to 4.448	3.381 to 3.632
HillSlope	-1.682 to -1.066	??? to -1.324	-1.360 to -0.9351	-3.266 to - 0.9645	-1.446 to -0.8476
IC50	5034 to 6805	1292 to 2384	4591 to 6585	13252 to 28023	2402 to 4284
**Goodness of Fit**					
Degrees of Freedom	5	3	8	7	8
R squared	0.9966	0.9886	0.9950	0.9665	0.9877
Sum of Squares	29.95	92.79	82.38	441.2	203.4
Sy.x	2.447	5.561	3.209	7.939	5.042
**Number of Points**					
# of X values	15	15	15	15	15
# Y values analyzed	7	5	10	9	10

Supplemental Table S2: Results for nonlinear regression analysis for neflamapimod and IL-1β-induced IL-6

Log(Inhibitor) vs. Normalized Response -- Variable Slope – Least Squares Fit	241204 1112	250204 101	250225 179
**Best-fit Values**			
LogIC50	3.750	3.954	3.658
HillSlope	-1.617	-1.276	-1.092
IC50 (pM)	5628	8992	4548
**95% CI (Profile Likelihood)**			
LogIC50	3.680 to 3.818	3.869 to 4.040	3.471 to 3.838
HillSlope	-2.118 to - 1.225	-1.647 to - 1.019	-1.775 to - 0.7399
IC50 (pM)	4784 to 6574	7399 to 10957	2960 to 6887
**Goodness of Fit**			
Degrees of Freedom	3	7	7
R squared	0.9977	0.9935	0.9726
Sum of Squares	16.65	93.43	361.6
Sy.x	2.356	3.653	7.187
**Number of Points**			
# of X values	9	9	9
# Y values analyzed	5	9	9

Table S3: Results for nonlinear regression analysis for neflamapimod and LPS-stimulated IL-1β

Log(Inhibitor) vs. Normalized Response - Variable Slope - Least Squares Fit	250109 5R	250204 100	250206 112	250225 178
**Best-fit Values**				
LogIC50	4.116	4.160	4.621	4.279
HillSlope	-0.6858	-0.7173	-0.7583	-0.7083
IC50	13076	14468	41809	19030
**95% CI (Profile Likelihood)**				
LogIC50	4.035 to 4.195	4.030 to 4.292	4.549 to 4.694	4.149 to 4.411
HillSlope	-0.7803 to -0.6043	-0.8858 to -0.5897	-0.8552 to -0.6751	-0.8661 to -0.5863
IC50	10830 to 15684	10727 to 19601	35416 to 49395	14096 to 25789
**Goodness of Fit**				
Degrees of Freedom	5	7	7	7
R squared	0.9968	0.9880	0.9959	0.9882
Sum of Squares	22.60	122.2	39.42	120.6
Sy.x	2.126	4.179	2.373	4.151
**Number of Points**				
# of X values	9	9	9	9
# Y values analyzed	7	9	9	9

Table S4: Results for nonlinear regression analysis for neflamapimod and LPS-stimulated TNF

Log(inhibitor) vs. Normalized Response - Variable Slope - Least Squares Fit	250107 5	250204 100	250206 112	250225 178
**Best-fit Values**				
LogIC50	4.760	4.909	5.546	5.194
HillSlope	-0.3479	-0.4626	-0.9869	-0.3710
IC50	57601	81056	351603	156376
**95% CI (Profile Likelihood)**				
LogIC50	4.423 to 5.093	4.661 to 5.188	5.412 to 5.694	5.009 to 5.407
HillSlope	-0.5083 to -0.2091	-0.6080 to -0.3478	-1.418 to -0.6920	-0.4461 to -0.3055
IC50	26515 to 123915	45822 to 154190	258429 to 493948	102141 to 255427
**Goodness of Fit**				
Degrees of Freedom	4	7	7	7
R squared	0.9318	0.9558	0.9654	0.9753
Sum of Squares	102.5	249.7	167.2	91.16
Sy.x	5.062	5.973	4.887	3.609
**Number of Points**				
# of X values	9	9	9	9
# Y values analyzed	6	9	9	9
